# Potential biological pathways linking Type-D personality and poor health: A cross-sectional investigation

**DOI:** 10.1371/journal.pone.0176014

**Published:** 2017-04-28

**Authors:** Vera K. Jandackova, Julian Koenig, Marc N. Jarczok, Joachim E. Fischer, Julian F. Thayer

**Affiliations:** 1 Department of Internal Medicine, Faculty of Medicine, University of Ostrava, Ostrava, Czech Republic; 2 Department of Human Movement Studies, Human Motion Diagnostic Centre, University of Ostrava, Ostrava, Czech Republic; 3 Department of Psychology, The Ohio State University, Columbus, Ohio, United States of America; 4 Section for Translational Psychobiology in Child and Adolescent Psychiatry, Department of Child and Adolescent Psychiatry, Centre for Psychosocial Medicine, University of Heidelberg, Heidelberg, Germany; 5 Institute of Medical Psychology, Center for Psychosocial Medicine, University Hospital Heidelberg, Heidelberg, Germany; 6 Mannheim Institute of Public Health, Social and Preventive Medicine, Mannheim Medical Faculty, Heidelberg University, Mannheim, Germany; Chiba Daigaku, JAPAN

## Abstract

**Background:**

Type-D personality, defined as a combination of high negative affect and high social isolation, has been associated with poor health outcomes. However, pathways underlying this association are largely unknown. We investigated the relationship between Type-D personality and several biological and behavioral pathways including the autonomic nervous system, the immune system, glucose regulation and sleep in a large, apparently healthy sample.

**Methods:**

Data from a total of 646 respondents (age 41.6±11.5, 12,2% women) were available for analysis. Persons with Type-D (negative affect and social isolation score ≥10) were contrasted with those without Type-D. Measures of plasma fibrinogen levels, white blood cell count, high sensitivity C-reactive protein, fasting plasma glucose (FPG), cholesterol, high-density and low-density lipoprotein, glycated hemoglobin (HbA1c), creatinine, triglycerides, and albumin were derived from fasting blood samples. Urine norepinephrine and free cortisol were determined by high-performance liquid chromatography. Time-domain heart rate variability (HRV) measures were calculated for the 24hr recording period and for nighttime separately.

**Results:**

Persons with Type-D had higher HbA1c, FPG, and fibrinogen, and lower nighttime HRV than those without Type-D, suggesting worse glycemic control, systemic inflammation and poorer autonomic nervous system modulation in Type-D persons. In addition, those with Type-D reported less social support and greater sleep difficulties while no group differences were observed for alcohol and cigarette consumption, physical activity and body mass index.

**Conclusions:**

Findings provide some of the first evidence for multiple possible biological and behavioral pathways between Type-D personality and increased morbidity and mortality.

## Introduction

The “distressed” Type-D personality is defined as a combination of high negative affect (NA) and high social isolation (SI)[1]. It has been distinguished from a number of related constructs such as depression, and vital exhaustion, and accounts for the interplay by which SI may modulate the association of NA with cardiovascular disease (CVD)[2]. Importantly, Type-D has been independently associated with various negative health outcomes in both cardiac and non-cardiac patients[3,4]. For example, a meta-analysis of prospective cohort studies showed a significant association between Type-D and mortality and nonfatal myocardial infarction. However, the authors suggest, that the association of Type-D and the prognosis in cardiac patients is different for the type of cardiac diseases[5]. Despite this plethora of studies, the pathophysiological mechanisms linking Type-D to adverse outcomes in CVD[5], and the biological pathways via which Type-D produces its deleterious effects are largely unknown.

A relatively few studies have investigated physiological or behavioral pathways underlying Type-D, such as markers of inflammation[6], hemoglobin[7], glucose and cholesterol levels[8,9], physical activity[10], or sleep[11]. Only some of these studies investigated samples of non-cardiac patients or healthy individuals[3]. While cross-sectional studies support an association of metabolic syndrome (METS) and Type-D[12], prospective studies do not[8]. In a population-based cohort Type-D was associated with fewer sleeping hours and an increased risk of sleep disturbances in adolescents[11]. However to date, no studies have examined several potential biological pathways and behavioral markers in a sample of non-cardiac patients.

Several experimental studies, have found Type-D personality associated with an exaggerated hemodynamic response to induced laboratory stress[13], suggesting autonomic nervous system (ANS) dysfunction as a mechanism linking Type-D to poor health. Heart rate variability (HRV) is a valid non-invasive technique to assess ANS function[14]. Low HRV, indicative of diminished vagal cardiovascular modulation, has been linked to increased risk of all-cause mortality and CVD[14–16]. ANS function, indexed by HRV, is further associated with inflammation[17], cholesterol levels[18], poor sleep[19], incident depression[20], self-rated health[21] and glucose regulation even after controlling for sympathetic activity [22]. While some preliminary studies found an association of HRV and Type-D in healthy subjects[23], and patients with an implantable cardioverter-defibrillator[24], HRV did not differ significantly between those with or without Type-D in non-cardiac patients[25].

Given the complex interplay of the ANS, the immune system, glucose regulation and associated health behaviors (e.g. disturbed sleep), conclusions drawn on single pathways in isolation should be drawn carefully. For instance, recent research provides evidence, that the interplay of ANS dysfunction and inflammation contribute to CVD-related mortality risk associated with depression[26]. Furthermore, most of the studies looking at the physiological pathways underlying Type-D are limited as they investigate either cardiac patients or student samples only. Therefore the present study was designed to investigate the relationship between Type-D personality—and its two psychological traits NA and SI—and several biological and behavioral pathways, including the autonomic nervous system, the immune system, glucose regulation and sleep, while controlling for health behaviors and various symptoms in a large, apparently healthy, occupational sample.

## Materials and methods

### General procedures

The study population comprised apparently healthy employees of an airplane manufacturing plant in Southern Germany. This occupational cohort was initiated in 2000 with additional recruitment and follow-up in 2002, 2003/2004 and 2007. For this analysis, we used cross-sectional data from September 2003 and February 2004, because HRV data was available only for the sample recruited during this period of time. In 2003/2004 questionnaire data were collected and medical examinations were carried out. This study was based on an open cohort approach meaning that follow-up assessments were not limited to the baseline population of 2000, but that this cohort was replenished with additional participants or volunteering employees at follow-up that had not participated at baseline. The sample spanned the entire age of the work force (18–63 years) and all levels of socioeconomic status (from the general manager to unskilled workers). The majority of the participants were engineers or highly skilled aircraft mechanics. A total of 657 individuals partook in the medical examination in 2003/2004 including a full day recording of heart rate. Of these, 11 participants with ECG recordings failure or missing data on Type D personality construct were excluded. We didn´t exclude participants with incomplete laboratory or other questionnaire data. This yielded a final sample of 646 participants. The study was approved by the institutional review board of the Federal Institute of Technology, Zurich, Switzerland. All participants signed informed consent prior to any examination.

After basic sociodemographic information and questionnaire data were collected basic medical examinations were carried out. Prior to the heart rate recording blood pressure via sphygmomanometry was recorded from the dominant arm. To assess 24h and nighttime heart rate and heart rate variability measures, study participants were instrumented with the ambulatory ECG recorders (Mini-Vitaport ECG logger, Becker Medical Systems, Karlsruhe, Germany, sampling rate 400Hz) between 9 and 12a.m. and were monitored until the next morning. Individuals proceeded with their usual work routine until 3.30 p.m. and then continued with their usual leisure and sleep activities. ECG recorders were disconnected at the next morning between 7:15 and 8:00 a.m. After returning the monitors fasting blood samples were collected to obtain measures of plasma fibrinogen levels, white blood cell count (WBC, leukocytes), C-reactive protein (CRP), fasting plasma glucose (FPG), cholesterol, high-density (HDL) and low-density lipoprotein (LDL), glycated hemoglobin (HbA1c), creatinine, triglycerides, and albumin. Blood samples were immediately transported to a laboratory (Synlab, Augsburg, Germany), for analysis within 6h of sample collection. To determine urine norepinephrine and urinary free cortisol urine collection was completed overnight from 9p.m. the night preceding the blood sample until and including the first voiding in the morning.

### Self-report questionnaires and health behavior

Type-D personality was measured with the DS14[1] self-report scale comprising 14 items, 7 items referring to NA and SI, respectively. Each item is rated on a 5-point Likert-scale (0–4 range). DS14 was found to adequately measure the personality traits NA and SI, with highest reliability in both traits at the cutoff of 10[27]. This cutoff was shown to be accurate and valid in classifying individuals as those with versus without Type-D both in general and clinical populations[27,28]. The Short Form Health Survey (SF12) was used to assess health related quality of life (HRQoL). The SF12 is a widely used standardized 12-item short form developed from the original Short Form–36 Health Survey[29,30]. It has proven to be a psychometrically robust and practical instrument in the outcome evaluation of subjective health functioning across different countries and populations[30]. For the present analysis, the SF-12 *physical score* and the *mental score* were calculated indicating overall physical and mental health status. Scale scores were standardized, with higher scores indicating better health status. The SF-12 is a reliable and valid instrument [3]. Symptoms of depression and anxiety were assessed with the 14-item Hospitality Anxiety and Depression Scale (HADS)[31], consisting of 2 subscales, with 7 items each, scored on 4-point Likert scales (from 0–3; subscale maximum score 21). A cut-off score of 8 and higher on each subscale represents clinically relevant levels of anxiety and depression [31]. The HADS has been proven a valid and reliable instrument to detect symptoms of anxiety and depression [31]. The average amount of cigarettes smoked per day was assessed and current smokers were defined as those who reported to smoke at least one cigarette per day. Alcohol consumption was measured as the cumulative number of alcoholic beverages (specified in equivalent units: e.g., bottle or can of beer 0.5l/unit; wine 0.2l/unit; liquor 50 ml/glass) consumed on average (response categories: not at all; 1-3/month; 1/week; 2-4/week; 5-6/week; 1/day; 2-3/day; 4-5/day; 6 or more/day), and further converted into average consumption in gr/day. Sleep problems were assessed using the Jenkins Sleep score [32], a widely used self- reported measure of sleep quality. The questionnaire comprises four items (e.g. “Have trouble falling asleep”, “Have trouble staying asleep”), where participants are requested to indicate how often in the past month they experienced each sleep problem. Items were rated on a six-point scale (ranging from 0 = “not at all”, to 5 = “22–31 days”). The scores were averaged, with higher scores corresponding to greater sleep problems (range 0–5). Data on physical activity was derived from a self-report questionnaire and assessed during the 24-hour ECG recording by means of an inbuilt 3D-accelerometer of which physical activity per kilogram was estimated.

### Physiological variables

Blood pressure (BP) was measured three times with 15-minute intermediate resting periods while the participants stayed seated. Mean systolic (SBP) and diastolic BP (DBP) were calculated as the mean of the second and the third measurements. Raw ECG data were processed according to the Task Force Guidelines[14] that define standards for the measurement, physiological interpretation, and clinical use of HRV. Inter-Beat-Intervals (IBIs) were calculated as the time between successive R-spikes. IBIs that corresponded to a mean heart rate (HR, beats per minute-Bpm) <30 or >200 as well as IBI changes of over 30% were excluded from further analysis (artifact correction). The mean HR and several time-domain measures of HRV were derived from the IBI time series for the 24h and nighttime only periods. The mean IBI (ms), the standard deviation of all inter beat intervals (SDNN, ms), the root mean square of successive differences (RMSSD, ms), and the percentage of differences between adjacent NN intervals differing by more than 50ms (pNN50, %) were calculated. The RMSSD was used as an index of vagally mediated HRV.

Measures of plasma fibrinogen levels, white blood cell count, C-reactive protein, fasting plasma glucose, cholesterol, high-density and low-density lipoprotein, glycated hemoglobin, creatinine, triglycerides, and albumin were derived from fasting blood samples. Plasma fibrinogen levels were determined by a routine clotting assay following the Clauss method[33]. WBC was determined on a Sysmex SE-9000 automated analyzer (Sysmex, Nordestadt, Germany). CRP was measured with a high-sensitivity assay (Dade Behring, Schwalbach, Germany). Urine norepinephrine and urinary free cortisol were determined by high-performance liquid chromatography. According to the U.S. National Library of Medicine [34] the normal values for physiological variables are as follows: 4.5 to 11.0μL for WBC count; 70 to 100 mg/dl for fasting plasma glucose; 5.7% for HbA1C; 0.7 to 1.3 mg/dl for creatinine in men and 0.6 to 1.1 mg/dl for creatinine in women; less than 150 mg/dl for triglycerides; 3.4 to 5.4 g/dl for albumin and 200 to 400 mg/dl for plasma fibrinogen. Desirable values are less than 190 mg/dl for cholesterol, less than 100 mg/dl for LDL; 60 mg/dl and higher for HDL and less than 0.1 mg/dl for CRP [34].

### Statistical analysis

We formed four groups based on the Type-D assessment: controls with neither NA nor SI trait score≥10 (n = 93); those with NA trait score ≥10 and SI score≤10 (n = 55); those with SI trait score≥10 and NA score≤10 (n = 208); and those with Type-D personality (NA and SI trait score≥10) (n = 290). Descriptive statistics including frequencies with percentages and means with standard deviations (SD) were calculated based on group comparisons. Planned contrasts examined differences between the four groups (controls|NA|SI|Type-D) on sociodemographic, health behavior, symptom distress and physiological variables. Specifically, the preplanned contrast–comparing those with Type-D to those without (-1|-1|-1|3)–was subjected to analysis to identify physiological and behavioral aspects uniquely associated with full Type-D. The planned contrasts were computed using one-way analysis of variance (ANOVA) and the results are reported using t-statistics. Statistical analyses were performed using STATISTICA (version 10, StatSoft). All tests were performed with a set α-level of 0.05 to indicate statistical significance. Intentionally, we have not corrected the p-values for multiple comparisons [35–37].

## Results

Data from a total of 646 respondents (mean age 41.6 years; 12,2% women) were available for analysis. The prevalence was 44.8% for full Type-D personality, 8.5% for negative affectivity (NA) trait and 32.1% for social inhibition trait. Sample characteristics on sociodemographic variables, health behavior and psychological symptom distress by group based on Type-D assessment are given in Table 1. Type-Ds were on average older than non-Type-Ds (combined group of controls and those with a single trait—NA or SI). We observed no significant group differences for life style factors, including alcohol and cigarette consumption, physical activity or BMI. As anticipated the full Type-D group was characterized by higher levels of negative affectivity and social inhibition compared with other groups combined. Results from other stress symptom measures indicated that the full Type-D group had lower scores on constructs reflecting lower subjective physical and mental health functioning and social support, higher levels of anxiety and depression and greater sleep difficulties than non-Type-D groups (p≤0.001).

**Table 1:** Sample characteristics I: sociodemographics, health behavior and symptom distress by group; contrast weights (-1/-1/-1/3).

									*Contrast*	
	Controls		NA trait		SI trait		Type D		*t*	*p*
n (%)	93	(14.4)	55	(8.5)	208	(32.1)	290	(44.8)		
Sex, *n (%)*										
*Women*	5	(5.7)	11	(21.2)	15	(7.8)	42	(15.8)		
*Men*	82	(94.3)	41	(78.8)	178	(92.2)	224	(84.2)		
Age, *years*	41	(11)	38	(13)	41	(11)	43	(11)	*2*.*390*	.*017*
Alcohol, *g per day*	14.31	(13.11)	16.66	(17.08)	15.61	(14.85)	18.26	(18.87)	*1*.*694*	.*091*
Current smoking, *n (%)*	24	(25.8)	20	(36.4)	53	(25.6)	77	(26.7)		
Physical activity per kg	4.39	(4.03)	3.69	(4.11)	3.94	(4.66)	4.22	(6.19)	*0*.*577*	.*56*
BMI, *kg/m*^*2*^	26.39	(3.20)	25.47	(3.52)	26.16	(3.80)	26.58	(4.37)	*1*.*284*	.*20*
Type-D: NA trait	5.38	(2.57)	12.64	(2.61)	6.78	(6.78)	14.50	(3.56)	*23*.*030*	*<* .*0001*
Type-D: SI trait	7.77	(1.31)	8.15	(8.15)	12.40	(2.02)	13.39	(2.49)	*23*.*031*	*<* .*0001*
Type-D: Total score	13.15	(3.13)	20.78	(2.91)	19.18	(3.03)	27.90	(4.90)	*27*.*337*	*<* .*0001*
SF-12: Physical score	53.12	(5.96)	51.60	(7.67)	52.11	(6.02)	47.31	(9.44)	*-7*.*157*	*<* .*0001*
SF-12: Mental score	53.41	(6.62)	42.17	(10.40)	52.07	(6.48)	43.07	(10.44)	*-7*.*389*	*<* .*0001*
HADS: Anxiety	4.08	(2.61)	7.75	(2.51)	5.04	(2.71)	8.11	(2.55)	*7*.*045*	*<* .*0001*
HADS: Depression	2.72	(2.18)	5.73	(3.02)	3.35	(2.43)	6.56	(3.10)	*10*.*065*	*<* .*0001*
Jenkins Sleep Index	3.33	(2.95)	6.78	(4.33)	4.11	(3.22)	7.18	(4.37)	*6*.*526*	*<* .*0001*
Social Support Scale	62.97	(5.99)	60.57	(8.22)	58.60	(8.67)	56.66	(9.04)	*-5*.*523*	*<* .*0001*

Note: Results are presented as means and standard deviations (SD), or frequencies n (%).

**Abbreviations:** BMI, body mass index; NA trait, negative affectivity trait; SI trait, social inhibition trait; SF-12, Short Form Health Survey; HADS, Hospitality Anxiety and Depression Scale.

Table 2 presents physiological aspects of the sample by group based on Type-D assessment. Contrasts indicated that the full Type-D group differed significantly from the non-Type-D groups combined in average levels of HbA1c (p = 0.020), fibrinogen (p = 0.006), nighttime SDNN (p = 0.003), pNN50 (p = 0.004) and RMSSD (p = 0.002). The contrast between full Type-D group and non-Type-D groups combined approached significance for FPG (p = 0.052). On average, full Type-D individuals had higher levels of HbA1c, FPG and fibrinogen and lower average levels of nighttime SDNN, pNN50 and RMSSD than non-Type-Ds. There were no statistically significant group differences in average levels of blood pressure, blood lipid markers (cholesterol, HDL, LDL, triglycerides), WBC, CRP, cortisol-creatinine ratio, norepi-creatinine ratio, albumin-creatinine ratio, creatinine, albumin, and 24-hour HR/HRV indices and nighttime HR and IBI.

**Table 2:** Sample characteristics II: physiological variables by group; contrast weights (-1/-1/-1/3).

					*Contrast*	
	Controls	NA trait	SI trait	Type-D	*t*	*p*
n (%)	93(14.4) 124.77(15.79) 82.04(11.81) 211(46) 53(11) 129.31(37.74) 5.74(1.38) 5.29(0.51) .14(0.16) 39.64(19.13) 15.02(6.76) 4.92(13.13) 279(43) 92(17) 145(85) 79.26(9.09) 796.74(100.60) 146.14(41.00) .145(0.10) 38.17(13.89) 62.71(7.67) 985.93(130.31) 104.88(34.94) .228(0.20) 48.06(25.85)	55(8.5) 119.12(15.07) 77.24(10.21) 211(39) 55(15) 128.27(32.46) 5.99(1.56) 5.34(0.47) .19(0.26) 38.98(18.27) 16.91(8.55) 4.28(5.15) 287(47) 88(9) 142(86) 79.82(10.10) 793.74(100.57) 149.16(44.91) .167(0.11) 41.23(14.75) 63.15(7.79) 979.34(128.75) 107.72(35.14) .271(0.18) 53.25(24.25)	208(32.1) 122.81(15.23) 80.27(10.54) 215(43) 55(13) 133.17(38.34) 5.78(1.51) 5.34(0.41) .20(0.36) 43.16(18.92) 16.44(8.41) 12.27(103.22) 293(64) 91(14) 149(118) 79.24(9.00) 797.83(93.10) 149.81(44.53) .161(0.12) 39.42(14.76) 63.35(8.42) 977.06(130.76) 102.52(33.16) .233(0.18) 46.78(21.76)	290(44.8) 123.32(14.54) 80.31(9.72) 215(43) 55(14) 132.23(39.18) 5.89(1.75) 5.42(0.61) .23(0.45) 43.06(18.72) 16.75(10.05) 4.20(10.24) 300(67) 93(18) 162(147) 77.70(9.32) 813.57(100.60) 147.19(42.59) .152(0.11) 38.47(14.31) 63.63(9.84) 975.47(140.41) 95.86(34.70) .199(0.17) 43.21(21.57)		
SBP (mmHg)					*0*.*795*	.*43*
DBP (mmHg)					*0*.*724*	.*47*
Cholesterin (mg/dl)					*0*.*797*	.*43*
HDL (mg/dl)					*0*.*698*	.*49*
LDL (mg/dl)					*0*.*577*	.*56*
WBC (μL)					*0*.*069*	.*53*
Glycosylated-hemoglobin					*2*.*339*	.*020*
Log-CRP (mg/dl)					*0*.*611*	.*54*
Cortisol/creatinine (urinary)					*1*.*521*	.*13*
Norepi/creatinine					*0*.*950*	.*34*
Albumin/creatinine					*-0*.*576*	.*57*
Fibrinogen					*2*.*781*	.*006*
Fasting plasma glucose (mg/dl)					*1*.*950*	.*052*
Triglcycerides (mg/dl)					*1*.*669*	.*096*
HR-24h (bpm)					*-1*.*735*	.*083*
IBI-24h (ms)					*1*.*627*	.*10*
SDNN-24h (ms)					*-0*.*789*	.*43*
pNN50-24h (%)					*-1*.*069*	.*29*
RMSSD-24h (ms)					*-1*.*417*	.*16*
HR-sleep (bpm)					*0*.*616*	.*54*
IBI-sleep (ms)					*-0*.*358*	.*72*
SDNN-sleep (ms)					*-3*.*023*	.*003*
pNN50-sleep (%)					*-2*.*869*	.*004*
RMSSD-sleep (ms)					*-3*.*125*	.*002*

Note: Results are presented as means and standard deviations (SD), or frequencies n (%).

**Abbreviations**: SBP, systolic blood pressure; DBP, diastolic blood pressure; HDL, high-density lipoprotein; LDL, low-density lipoprotein; WBC, white blood cell count; log CRP, log-transformed C-reactive protein; HR, heart rate; IBI, inter beat interval; SDNN, the standard deviation of all inter beat intervals; pNN50, the percentage of differences between adjacent NN intervals differing by more than 50ms; RMSSD, the root mean square of successive differences.

## Discussion

We examined multiple biological and behavioral markers in association with Type-D personality in a large, apparently healthy, occupational sample. We found that men and women with Type-D personality had higher HbA1c, FPG, fibrinogen and lower nighttime vagally mediated HRV indices than those without Type-D personality. This may indicate worse glycemic control, systemic inflammation and poorer ANS modulation in individuals with Type-D personality compared with those without Type-D. In addition, men and women with Type-D personality reported greater sleep difficulties and lower social support. Importantly these results are not due to life style factors as we observed no significant group differences in alcohol and cigarette consumption, physical activity and BMI. The novelty of this study lies in demonstrating complex biological and behavioral profiles in apparently healthy Type-D individuals which may help to understand the plausible mechanisms linking Type-D personality with negative health outcomes.

The present study found that Type-D personality is associated with markers related to worse glucose regulation[8,9], as reflected by higher HbA1c and FPG, and is not associated with other MET factors, such as BP, blood lipids or BMI. These findings are consistent with cross-sectional results from a German occupational cohort, where plasma glucose was significantly higher in Type-Ds compared with non-Type counterparts and no differences were observed in BP, triglycerides and BMI[8]. In our study, the magnitudes of observed group differences for glucose regulation accords with this earlier work[8] and suggest rather small differences (t = 2.3 for HbA1c and t = 2.0 for FPG). However it is well established that even small changes in glucose regulation have significant implications for diabetes and CVD risk[38–40]. For example, a large epidemiological study showed that an increase in Hb1Ac of 1% point was associated with 24–28% increase in CVD events and all-cause mortality for both men and women[38]. HbA1c may be of particular importance as it reflects long-term (2–3 months) glucose concentration and has been shown to be a better predictor of microvascular diseases than FPG[40].

The observed increased level of fibrinogen, although in the normal range (200-450mg/dl), may reflect higher inflammation and coagulation. Increased levels of fibrinogen may indicate inflammatory vascular changes and endothelial dysfunction[41]. Increases in plasma fibrinogen levels of 1g/liter, even in the normal range, have been found to be associated with twice the risk of major CVD outcomes[42]. No prior study has investigated fibrinogen in association with Type-D but platelet activation and higher levels of fibrinogen have been shown to be increased during emotional stress[43]. In the present study, we observed no group differences on other inflammatory markers (CRP or WBC). The results for CRP are in agreement with two previous studies in CHF and non-cardiac individuals[6,44].

The specific associations of Type-D personality with indices of nighttime HRV, not 24hour HRV, may reflect worse vagal modulation of the heart in Type-Ds, since there is nocturnal predominance of vagal activity. Only few studies have examined HRV in association with Type-D but the findings are mixed[23–25]. The discrepancies in results may be due to different study procedures, populations and length of HRV recordings. In our study, individuals with Type-D reported worse sleep quality which is in agreement with prior evidence[11]. Insufficient sleep quality has been associated with lower HRV[19]. Additional contrast analysis comparing nighttime HRV in Type-Ds and non-Type-Ds while adjusted for sleep quality revealed largely similar results (p≤0.025). This leads to the conclusion that sleep quality does not account for the HRV-Type-D association. In sum, findings suggest that Type-D is associated with indices related to worse glucose regulation, systemic inflammation and lower vagal modulation.

Vagal modulation of the ANS is of particular importance as it may represent a direct link between the Type-D personality and less favorable health outcomes. The vagus nerve innervates a wide range of organ systems in the body and therefore provides information about the state of the organism to the brain. In previous research both coagulant responses and glycemic control have been shown to be associated with ANS modulation, particularly with vagally-mediated HRV[22,45]. For example, the vagus nerve has been shown to exert a tonic inhibitory control on pro-inflammatory cytokine production and to contribute to many inflammation-related diseases, including CVD[46]. Similarly vagal afferents in the hepatic portal provide information about the peripheral glucose status to the brain and vagal efferent fibers innervate the liver and the kidneys and play a role in glucose regulation[47]. Besides the importance of the vagus nerve in transmitting information concerning immune status and blood glucose to the brain, of particular relevance to this study, the autonomic nervous system, measured by HRV, has been shown to be involved in emotional regulation and dysregulation[24,48]. For example, individuals with lower resting HRV produce less context appropriate emotional responses[48]. Additionally lower HRV has been associated with social threat and social anxiety[49]. As suggested in previous work, affective and social behavior may be closely related to the ability to regulate visceral homeostasis, including control of the heart, mediated via vagal signaling[50]. Higher negative affectivity and social inhibition prevalent in Type-D individuals may therefore represent lower ability to regulate visceral homeostasis, as reflected by higher HbA1c, FPG, fibrinogen and lower nighttime vagally-mediated HRV indices in Type-Ds in the present study. Decreased ability to self-regulate and respond appropriately to environmental changes or stressors increases vulnerability to damage that may in the long run contribute to the increased risk of CVD and mortality, often reported in Type-Ds[2–6].

Our results of less favorable biomarkers, in men and women with “distressed” personality may be explained in light of the model of *Neurovisceral Integration* of cardiac vagal control[50]. In this model, adaptation to environmental challenges is shaped by influences from many sources: physiological, behavioral, cognitive, affective, social and environmental. Indeed, meta-analysis of neuroimaging studies[50] have shown low HRV to be associated with decreased blood flow in specific brain regions, including the amygdala and ventromedial prefrontal cortex (PFC), involved in perceptions of threat and safety and in regulation of negative emotion. In safe contexts, fear or threat representations in the amygdala, a rapid detector of potential threats[51], appear to be inhibited by the prefrontal cortex and ventromedial PFC (vmPFC) in particular. Furthermore, frontal cortex structures are particularly involved in visceral sensory processing[52] and linked with immune and glucose regulation. For example, higher circulating glucose levels were shown to be associated with greater activity in the medial PFC[53]. VmPFC may therefore be a specific brain region where the complete information is integrated and processed. Additionally, in the neurovisceral integration model[50], the communication between the peripheral nervous system and the brain is bi-directional. Our study is cross-sectional, making the exact understanding between Type-D and less favorable biological profile and any possible cause-effect pathway difficult. However previous research has suggested that decreased HRV and vagal modulation is a risk factor for both physical and psychological negative health outcomes. The vagal pathway could therefore be an intervention target in Type-D. For instance, it has been shown that enhancing vagal cardiac modulation by physical activity or vagal stimulation, have favorable effects on HRV, emotions and cardiac health[54,55].

One of the possible mechanisms linking Type-D with reduced HRV indices, increased HbA1c, FPG and fibrinogen may be unhealthy behavior, such as smoking habits, alcohol consumption, diet or physical inactivity[10]. However, there were no differences between those with and without Type-D in alcohol and cigarette consumption, physical activity or BMI, indicating that there may be an independent effect of Type-D apart from adverse life style.

The prevalence of 44.8% for Type D personality in our study was found to be higher than that reported in other European countries. Previous studies have identified rates of between 21% and 38.5% elsewhere in Europe[1,56,57]. For example 21% in Holland[1], 28% in Italy[56] and 38.5% in UK and Ireland[57]. In a population based cohort study in the Rhine-Main region (n = 5000), also located in the southern part of Germany, the prevalence rate of Type D was about 22.2%[58]. The reason of higher prevalence of Type D personality in our study may be due to the characteristics of the sample associated with a type of occupation. However in the Beutel´s et al.[58] study there is no information about work characteristics including how many are retired and not working therefore difficult to compare. Other two potential sources for the differences may be the proportion of men and women in the samples and the average age: In the Beutel´s et al.[58] sample there are many more females and the average age is much higher than in our sample (55 vs. 41 years).

### Strengths and limitations

A significant limitation of the current study is the cross-sectional design that prevents any assessment of causality and use of self-reported measures to assess life-style factors such as sleep quality and habitual physical activity. The sample is an occupational-based cohort and composed largely of Caucasian men therefore our data are not well generalizable especially to women and other ethnicities. We have not accounted for potential covariates such as prevalence of cardio-metabolic or mental illness, and/or medication use in the statistical analyses. However based on previous work using this sample there are only very few people with CVD and/or diabetes. In addition these factors were shown to have no effect on results in our previous work[18]. Thus our sample is considered apparently healthy. The key strengths of this study include multiple biomarkers and a large sample size. Findings are not limited to one particular professional group as the sample comprised of employees with differing socioeconomic backgrounds from four distinct geographical regions within Germany. Finally HRV was recorded over 24h.

## Conclusions

In conclusion men and women with Type-D personality, characterized by high NA and SI, exhibited higher HbA1c, FPG, fibrinogen and lower nighttime vagally-mediated HRV than non-Type-D counterparts. This suggests that Type-D is associated with biomarkers related to worse glycemic and ANS regulation as well as inflammation. Additionally, men and women with Type-D reported worse sleep quality and lower social support. Findings may illustrate potential mechanisms linking Type-D personality with increased morbidity and mortality. These may have implications for both prevention and treatment. First, physiological screening may be useful as a strategy to identify distressed individuals in order to prevent negative health outcomes. Second, targeting physiological and behavioral well-being may be effective in the treatment of distressed individuals. These and other potential implications should be examined in future research.
